# LncRNA HCG11 promotes proliferation and migration in gastric cancer via targeting miR-1276/CTNNB1 and activating Wnt signaling pathway

**DOI:** 10.1186/s12935-019-1046-0

**Published:** 2019-12-26

**Authors:** Hua Zhang, Haitao Huang, Xiaomei Xu, Haiying Wang, Jianxiang Wang, Zuoyi Yao, Xiaoyan Xu, Qian Wu, Fenlan Xu

**Affiliations:** 1 Department of Gastroenterology, The Fifth People’s Hospital of Chengdu, No. 33 Mashi Street, Wenjiang District, Chengdu, 611130 China; 2 Department of Respiratory, The Fifth People’s Hospital of Chengdu, No. 33 Mashi Street, Wenjiang District, Chengdu, 611130 China; 3 Department of Gynecology, The Fifth People’s Hospital of Chengdu, No. 33 Mashi Street, Wenjiang District, Chengdu, 611130 China; 4 Department of General surgery, The Fifth People’s Hospital of Chengdu, No. 33 Mashi Street, Wenjiang District, Chengdu, 611130 China; 5 Department of Anesthesiology, Chengdu Women’s & Children’s Central Hospital, Chengdu Riyue Avenue 1617, Chengdu, 610091 China; 6 Department of Anesthesiology, Chengdu Public Health Clinical Medical Center, Jingming Road 377, Chengdu, 610066 China

**Keywords:** HCG11, miR-1276, CTNNB1, Gastric cancer

## Abstract

**Background:**

Gastric cancer (GC) is one common cancer which occurs in the stomach leading to high mortality around the world. Long non-coding RNAs (lncRNAs) were found overexpressed or silenced in the occurrence and progression of multiple cancers including GC.

**Method:**

The gene expression level in GC tissues and cells were analyzed by RT-qPCR. CCK-8, colony formation, flow cytometry and transwell assays were performed for the function analysis of HLA complex group 11 (HCG11). The mechanism study for HCG11 was conducted using RIP, RNA pull down and luciferase reporter assays.

**Results:**

HCG11 was discovered highly expressed in GC tissues and cells. Depletion experiments were used to evaluate HCG11 silence on cell proliferation, migration and apoptosis. Moreover, Wnt signaling pathway was found as a tumor promoter in GC. RIP assay, RNA pull down assay and luciferase reporter assay were performed to illustrate the relationship of HCG11, miR-1276 and CTNNB1. Rescue assays revealed that HCG11/miR-1276/CTNNB1 axis regulated the incidence and development of GC. Tumor formation in mice proved that HCG11 was negatively correlated with miR-1276 and had positively correlation with CTNNB1.

**Conclusion:**

Overall, HCG11 accelerated proliferation and migration in GC through miR-1276/CTNNB1 and Wnt signaling pathway, revealing that HCG11 could be a brand new target for GC.

## Background

As one high death-rated cancer, gastric cancer brought fear and suffering to human’s physical and psychological health [1]. GC was marked with poor prognosis and lacked of effective treatment except intolerable chemotherapeutic accesses which undermine health. LncRNAs are a clan of RNAs whose length reach to over 200 nucleotides and have no ability of coding proteins. The abnormal expression of lncRNAs would result in the acceleration or deceleration in the process of cancer. Thanks to the massive achievements made in biology technology, emerging lncRNAs have been discovered to function as the anti-tumors or promoters in various cancers [2–4]. The association between lncRNA and GC was uncovered. For instance, LINC00460 was found promote cell proliferation and migration via targeting miR-342-3p [5]. HIT000218960 accelerated cell proliferation and migration by increasing expression of HMGA2 in GC [6]. However, the role of HCG11 in GC lacked enough evidence.

Wnt signaling pathway was one common-sighted signaling pathway closely related to diverse cancers [7–9]. It was known that CTNNB1 is the key player of the Wnt signaling pathway. Under normal conditions, it is not accumulated in the nucleus, where it could play important role in activating the transcription factors of the TCF/LEF family, leading to activation of genes responsive to Wnt. CTNNB1 could be translated into protein of β-catenin. β-catenin is the crucial protein symbolizing the activation of Wnt signaling pathway. Under normal conditions, it is situated in cytoplasm at a low expression level together with GSK3β multi-protein complex. However, when GSK3β is inactivated, β-catenin could transfer into nucleus and consequently alter the expression of target genes co-existed in nucleus [10]. In our study, western blot assay were applied to test the protein of key component parts of some common signaling pathways after transfecting with sh-HCG11#1. The results showed that β-catenin was prominently down-regulated in GC cells, suggesting Wnt signaling pathway may be involved in GC regulated by HCG11. CTNNB1 could be encoded into β-catenin. Hence, we presumed that HCG11 may work as a ceRNA to affect the expression of CTNNB1. MiRNAs were a group of RNAs with a length about 18–25 nucleotides. They own the capacity of preventing target mRNA coding into protein via binding to the 3′ untranslation region. Accumulating evidence suggested that miRNAs played a vital role in the progression of cancers [11, 12]. In our study, by means of starBase database, we selected out 19 miRNAs which had binding sites to both HCG11 and CTNNB1. Through further studies, miR-1276 was selected out to do the subsequent experiments. More details about the relationship between HCG11, miR-1276 and CTNNB1 needed more explanations.

This study centered on the function of HCG11 in GC cells. The results manifested that HCG11 promoted the progression of GC by targeting miR-1276/CTNNB1 and activating Wnt signaling pathway, which offered a novel insight for GC treatment.

## Materials and methods

### Human tissue samples

We collected 47 GC tissues from the Fifth People’s Hospital of Chengdu between 2013 and 2018. All patients did not accept any therapy before collection. Samples were preserved in liquid nitrogen. Our study gained permission and was approved by the Ethics Committee of the Fifth People’s Hospital of Chengdu. Written informed consent was signed by every participant. Experiments involving human tissues were performed in strictly line with the Declaration of Helsinki.

### Cell culture

Normal gastric cell (SNU-1) and gastric cancer cells (AGS, BGC-823, SGC-7901, SWMGC-803480) were bought from Chinese Academy of Sciences (Beijing, China). The cell culture procedure has been performed before [13]. Cells were preserved in DMEM (Invitrogen, Carlsbad, CA, USA) containing 10% FBS and 1% penicillin streptomycin (Sigma-Aldrich, Milan, Italy) at 37 °C with 5% CO_2._

### Cell transfection

Cells were put into 6-well plates until their density was 70–80% confluence. Then, the short hairpin RNAs (sh-RNAs) against HCG11 (sh-HCG11#1/2) and their negative control (sh-NC) were obtained from Genechem (Shanghai, China). The mimics and inhibitor of miR-1276, NC mimics and NC inhibitor were constructed by Genepharma. The above plasmids were separately transfected into AGS or BGC-823 cells using Lipofectamine 2000 (Invitrogen, Carlsbad, CA, USA). At 48 h after transfection, cells were gathered.

### Quantitative real-time PCR

In order to monitor the relative expression of RNA from cells or tissues, this experiment was conducted [14]. Below were primers utilized in this study: HCG11 primers forward: 5′-GCTCTATGCCATCCTGCTT-3′ and reverse: 5′-TCCCATCTCCATCAACCC-3′; miR-1276 primers forward: 5ʹ-TAGGTAAAGAGCCCTGTGGAGA-3ʹ and reverse: 5′-CATCAAGGCCCAAGTGCTCAG-3ʹ; CTNNB1 primers forward: 5ʹ-GCTGACCAAACTGCTAAATGACGA-3ʹ and reverse: 5ʹ-TGTAGGGTCCCAGCGGTACAA-3ʹ.

### Cell viability and colony formation assay

The purposes of these two assays were to test the proliferation ability of cells [15].

### Apoptosis assay

The apoptosis of AGS and BGC-823 cells was detected by using the Annexin V-APC Apoptosis kit (Multi Sciences, Shanghai, China), following the supplier’s direction. In short words, cells were cleaned and re-suspended with PBS (Solarbio, Beijing, China). Thereafter, cells were incubated with Annexin V-FITC for 15 min at 37 °C. Finally, flow cytometry was employed to detect the apoptosis of AGS and BGC-823 cells via measuring the fluorescent intensity.

### Migration assay

The aim of migration assay was to test the migration ability of cells. The top transwell chamber was applied with serum-free medium and the lower chamber was filled with 10% FBS (Solarbio). Moreover, cells were plated on the upper chamber of the membrane pre-coated without Matrigel for migration assay. After 48 h, migrated cells were fixed with methanol (Solarbio), and dyed with crystal violet (Solarbio). The images of five fields at random were captured via ×200 microscope (Olympus, Tokyo, Japan).

### Western blot

Total protein was prepared from GC tissues and cells using RIPA buffer, and protein quantification was performed by a spectrophotometer (Thermo Fisher Scientific, Carlsbad, USA). The extracts of protein were subjected to SDS-PAGE, followed by move to PVDF (Millipore, Bradford, MA, USA). After being blocked with nonfat milk, membranes were probed with primary antibodies at 4 °C overnight, and then incubated with secondary antibody for 1 h at dark room. Primary antibodies were as follows: anti-ß-catenin (ab32572, Abcam, Cambridge, USA), anti-GLI1 (ab49314, Abcam), anti-P13K (ab53610, Abcam), anti-AKT (ab81283, Abcam), anti-CTNNB1 (ab32572, Abcam) and anti-GAPDH (ab181602, Abcam), antibody against E-cadherin (ab40772), N-cadherin (ab76057), Vimentin (ab8978, Abcam), Twist (ab187008). The quantification of protein was analyzed by chemiluminescence system (GE Healthcare, Chicago, USA).

### TOP/FOP-Flash luciferase reporter analysis

TOP/FOP-Flash luciferase reporter assay was applied to analyze the activity of Wnt/β-catenin signaling pathway [16].

### Luciferase reporter assay

The wild-type and mutant binding sites of miR-1276 in the sequence of HCG11 were sub-cloned into pmirGLO dual-luciferase vector and constructed as HCG11-WT or HCG11-Mut plasmids respectively. Similarly, the wild-type and mutant binding sites of miR-1276 in CTNNB1 3′UTR were sub-cloned into pmirGLO dual-luciferase vector to construct plasmids, named CTNNB1-Wt or CTNNB1-Mut. Later, the reporter plasmids were co-transfected with miR-1276 mimics into AGS or BGC-823 cells. Ultimately, the luciferase activity was detected via the luciferase activity was detected via Dual-Luciferase Reporter Assay System (Promega, Massachusetts, USA). Firefly luciferase activity and renilla luciferase activity served as internal control.

### Subcellular fractionation

Cytoplasmic and Nuclear RNA Purification Kit (Norgen, Ontario, Canada) was employed for isolating and purifying cytoplasmic and nuclear RNA based on the specification. GAPDH and U6 were used as cytoplasmic or nuclear control respectively. Expression levels of HCG11 were assessed by RT-qPCR analysis.

### RNA pull down assay

The miR-1276-WT, miR-1276-Mut and NC were biotin labeled into Bio- miR-1276-WT, Bio-miR-1276-Mut and Bio-NC. Then, the biotinylated RNA was incubated overnight with cell lysate and then RNA-bound beads adhered streptomyces affidins were added. Finally, the purified RNA compound was tested by RT-qPCR.

### RNA immunoprecipitation assay

The RNA immunoprecipitation assay was completed in accordance with a previous report [17].

### Tumor formation in nude mice

Nude mice were bought from the Experiments Animal Center of Nanjing Biological Institute (Nanjing, China) and were stored in specific conditions. 10 mice were randomly fall into two groups. Tumor volume and weight were measured at specific time. After injection, mice were killed at the 28th day, and the tumors were removed, photographed.

### Fluorescence in situ hybridization assay

HCG11 subcellular localization was evaluated using a FISH Kit (Roche, Basel, Switzerland). GC cells were fixed with using paraformaldehyde (Solarbio). Then, a HCG11 probe (Sigma‐Aldrich) hybridization solution cultured in digoxigenin was added into the plate. An antagonistic HCG11 probe was constructed and regarded as NC. The 4′,6‐diamidino‐2‐phenylindole (Sigma‐Aldrich) was applied to stain nucleus for 10 min. Lastly, a laser confocal scanning microscopy (Olympus) was used to collect the fluorescence images.

### Immunohistochemistry assay

Excised tumor and gastric tissues were fixed in 4% paraformaldehyde, dehydrated and embedded with paraffin, finally cut into tumor sections. Consecutive 4-μm-thick tumor sections were immunostained with specific primary antibodies targeting E-cadherin (ab76055), N-cadherin (ab202030), Bax (ab81083) and bcl-2 (ab196495) from Abcam as per the established protocol.

### Statistical analysis

Data were expressed as mean ± standard deviation (SD). GraphPad Prism 7 software package (Graph-Pad Software, La Jolla, CA, USA) or SPSS (SPSS, Chicago, IL, USA) was used to analyze the experimental data. ANOVA was used to confirm the difference of multiple groups and Student’s t-test was employed for two groups. The gene expression correlation was detected by Pearson’s correlation analysis. P < 0.05 had statistically significance. And this experiment was done in triplicate.

## Results

### HCG11 was in high level of expression in GC tissues and cells

**Fig. 1:**
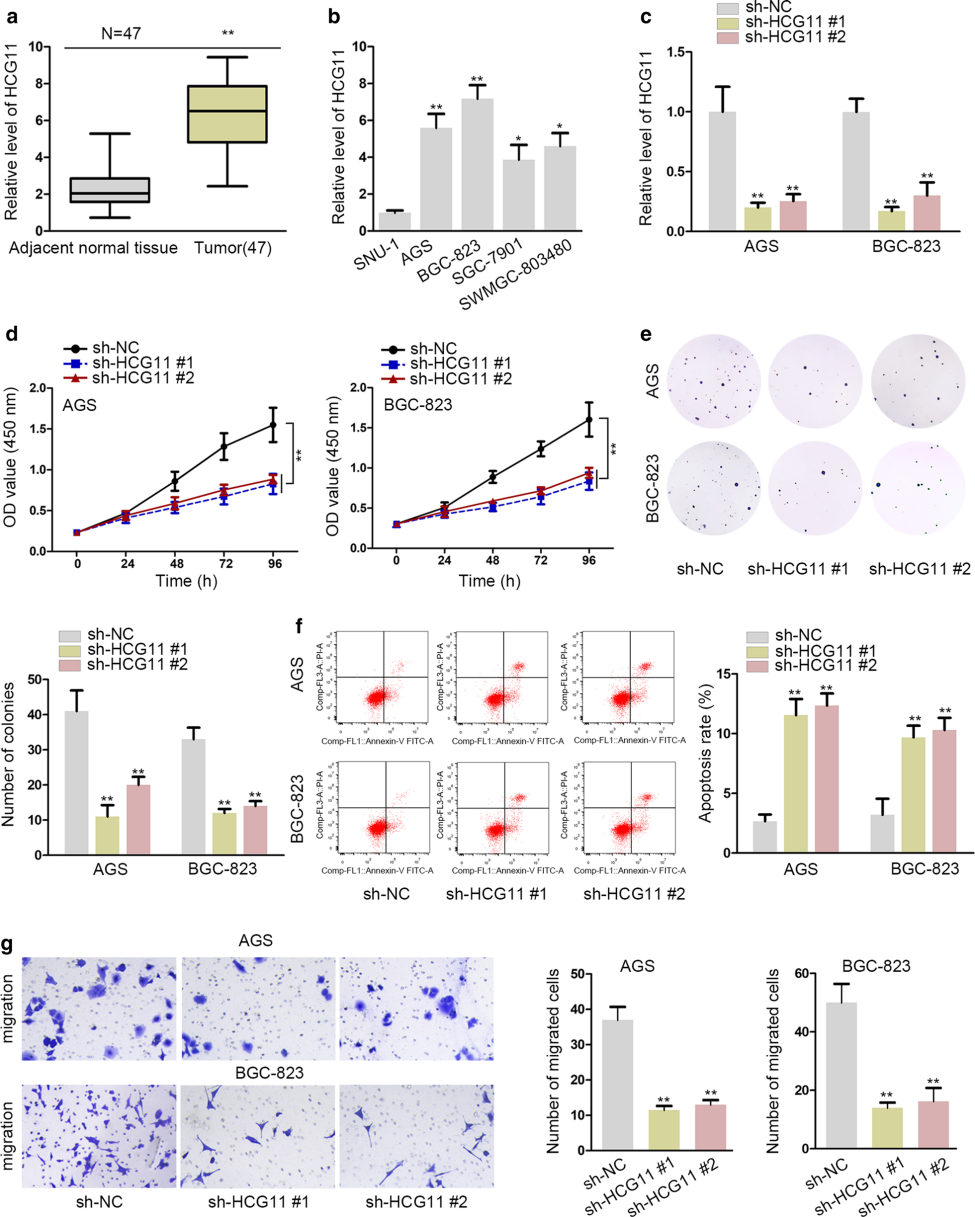
HCG11 was in high level of expression in GC tissues and cells. **a** RT-qPCR assays were carried out to test HCG11 expression in 47 pairs of GC tissues and adjacent normal tissues. **b** The expression of HCG11 was detected by RT-qPCR assay in GC cell lines (AGS, BGC-823, SGC-7901 and SWMGC-803480) and normal stomach cell (SNU-1). **c** The expression of HCG11 in GC cells was reduced by sh-HCG11#1, #2. **d**, **e** CCK8 assay and colony formation were performed to examine ability of cell proliferation. **f** Flow cytometry analysis was carried out to test apoptosis rate. **g** Transwell assay was conducted to assess capacity of migration. *P < 0.05, **P < 0.01

HCG11 was a common lncRNA found in various cancers. To determine its role in GC, we test the expression of HCG11 among 47 pairs of GC tissues and adjacent normal tissues. The outcome presented that HCG11 was highly expressed in GC tissues (Fig. 1a). Besides, we also found that HCG11 was significantly abundant in advanced stages of gastric (III + IV) compared with that in gastric cancer early stages (I + II), indicating the crucial role of HCG11 in the development and progression of gastric cancer (Additional file 1: Figure S1A). Then, RT-qPCR assays were applied to evaluate HCG11 expression in GC cells lines (AGS, BGC-823, SGC-7901 and SWMGC-803480) and normal stomach cells (SNU-1) (Fig. 1b). AGS and BGC-823 cells were transfected with sh-HCG11#1, sh-HCG11#2 and accessed their efficiency by RT-qPCR assay. The outcomes revealed that the expression of HCG11 was successfully turned down (Fig. 1c). Functional assays were carried out to detect the ability of cell proliferation, apoptosis and migration in GC cells lines transfected with sh-HCG11#1, #2. The results of CCK8 assay and colony formation assay delineated that the ability of GC cell proliferation was decreased (Fig. 1d, e). The rate of apoptosis was increased by knockdown of HCG11 in flow cytometry analysis (Fig. 1f). The capacity of migration tested by transwell assay was impaired by HCG11 silence in GC cells (Fig. 1g). Additionally, western blot assay was performed to evaluate the level of proteins related with epithelial–mesenchymal transition after silencing HCG11. We observed that knockdown of HCG11 overtly increased the level of E-cadherin, while decreasing those of N-cadherin, Vimentin and Twist (Additional file 1: Figure S1B). These results further validated the anti-migration effects induced by silencing HCG11. Taken together, the above results revealed that HCG11 was highly expressed in GC and knockdown of HCG11 hindered the GC cell proliferation and migration, yet accelerating apoptosis.

### HCG11 accelerated the proliferation and migration in GC cells by activating Wnt signaling pathway

**Fig. 2:**
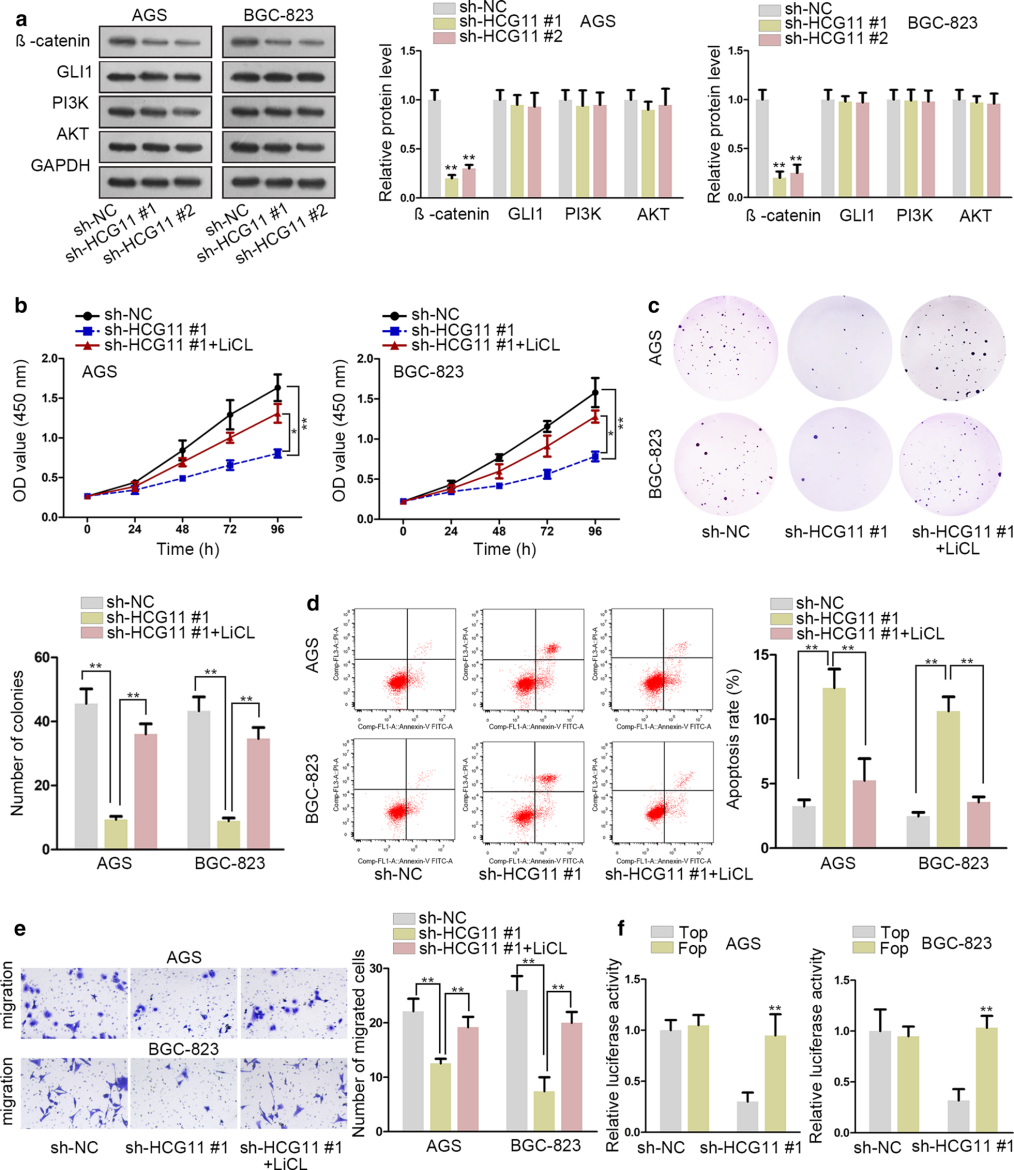
HCG11 accelerated the proliferation and migration in GC cells by activating Wnt signaling pathway. **a** Western blot was applied to measure key proteins of several signaling pathways. **b**, **c** Cell proliferation ability was decreased by knockdown of HCG11, examined by CCK8 and colony formation assay, which could be recovered by LiCl. **d** The rate of apoptosis was increased by sh-HCG11#1, which could be regained by LiCl. **e** The capacity of migration was impaired by sh-HCG11#1, which could be counteracted by LiCl. **f** TOP/FOP flash luciferase reporter assay was conducted to test the activity of signaling pathway. *P < 0.05, **P < 0.01

Accumulating studies hinted that signaling pathway was closely connected to the occurrence and development of cancers. Thus, we guess one signaling pathway may take part in the process of GC. Western blot assay was used to detect the key components of several common sighted signaling pathways, such as β-catenin, GLI1, PI3K and AKT. The results presented that the expression of β-catenin, which was a crucial part of Wnt signaling pathway, decreased dramatically while no distinct change could be found in other key proteins connected with Hedgehog and PI3K/AKT signaling pathway (Fig. 2a). That means, Wnt signaling pathway might be involved in GC progression. Additionally, the sh-HCG11#1 transfected cells were treated with Wnt signaling activator LiCl. The proliferation ability in GC cells was weakened by sh-HCG11#1, while enhanced again after treating with LiCl (Fig. 2b, c). Meanwhile, the apoptosis capability was enhanced after silencing HCG11, but impaired again after treating with LiCl, as illustrated in Fig. 2d. More importantly, the migration ability inhibited by sh-HCG11 was restored again after LiCl treatment (Fig. 2e). TOP/FOP flash luciferase reporter assay revealed that down-regulated HCG11 dramatically declined the activity of Wnt signaling pathway (Fig. 2f). These results demonstrated that HCG11 was involved in the activation of WHT signaling pathway.

### HCG11 sponged miR-1276 to regulate CTNNB1

**Fig. 3:**
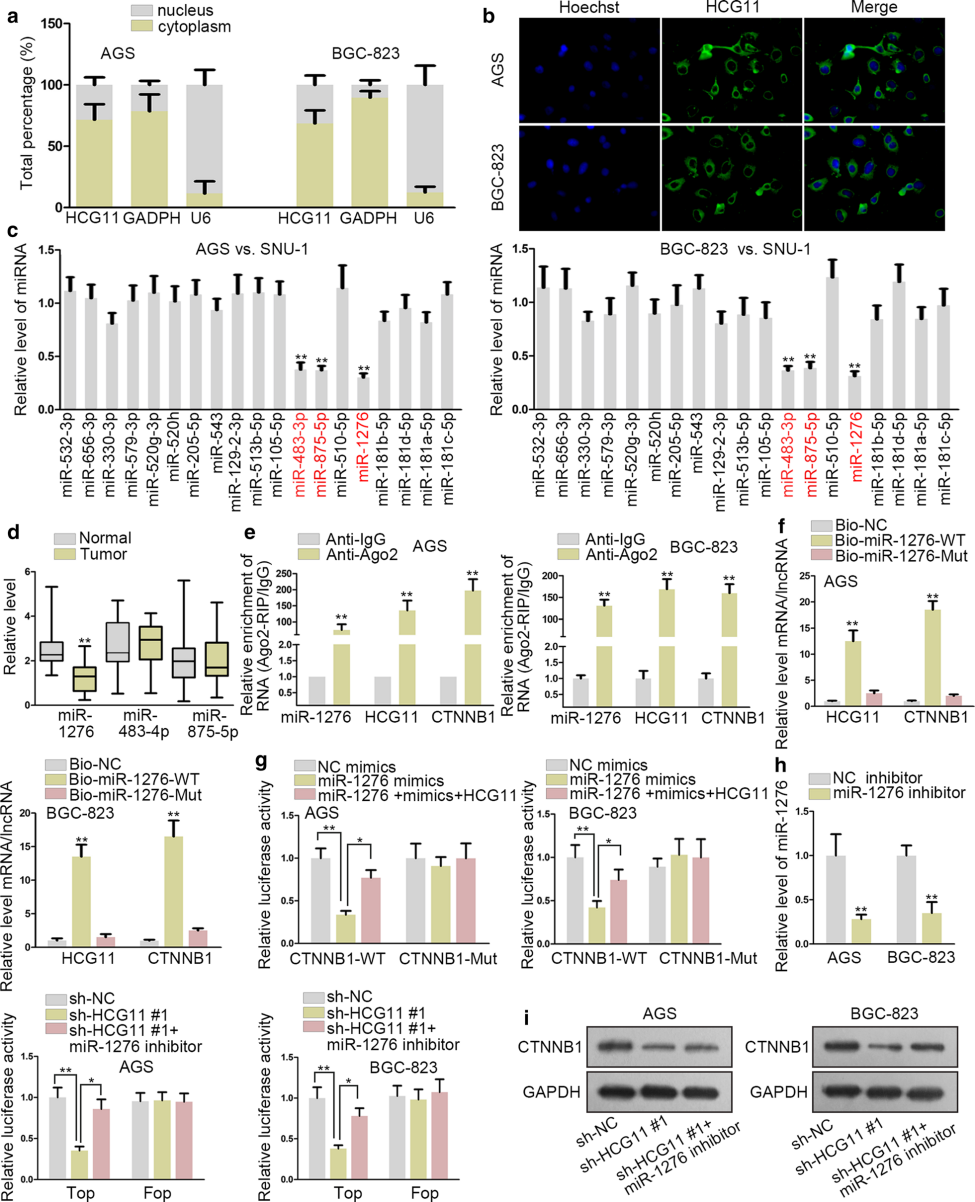
HCG11 sponged miR-1276 to regulate CTNNB1. **a**, **b** Nuclear-cytoplasmic fractionation and FISH assay were conducted to illustrate that HCG11 was mainly in cytoplasm. **c** RT-qPCR assays were used to examine the expression of 19 miRNAs in GC cells. **d** MiR-1276, miR-483-4p and miR-875-5p expression was detected in 47 pairs of GC tissues and normal adjacent tissues. **e** RIP assay was applied to test the mechanical relationship of HCG11, miR-1267 and CTNNB1. **f** RNA pull down assay was used to demonstrate whether miR-1276 could bind to HCG11 and CTNNB1. **g** Luciferase reporter assay was conducted to verify competing relationship among HCG11, miR-1276 and CTNNB1. **h** TOP/FOP flash luciferase reporter assay was used to examine the activity of signaling decreased by sh-HCG11, which was counteracted by miR-1276 inhibitor. **i** Western blot assay was used to detect protein of CTNNB1 decreased by silencing HCG11, which was recovered by miR-1276 inhibitor. *P < 0.05, **P < 0.01

To investigate the detailed function involved in GC cells, firstly, Nuclear-cytoplasmic fractionation was used to explore the subcellular localization of HCG11. The outcome indicated that HCG11 mainly accumulated in cytoplasm (Fig. 3a). Subsequently, FISH assay conducted further proved that HCG11 was mainly amassed in cytoplasm (Fig. 3b). Therefore, we assumed that HCG11 might act as a ceRNA to activate Wnt signaling pathway. CTNNB1 was the mRNA which could be coded and translated into protein of β-catenin. We presumed that HCG11 mediated one miRNA to regulate the expression of CTNNB1. With the help of starBase website (http://starbase.sysu.edu.cn/), 19 miRNAs which could bind to both HCG11 and CTNNB1 were selected out. The results of RT-qPCR assay showed that only the expression of miR-483-3p, miR-875-5p and miR-1276 declined prominently in GC cell lines (Fig. 3c). Next, the expressions of those three miRNAs were detected in 47 pairs GC tissues and normal adjacent stomach tissue. The outcome revealed that miR-1276 expression was significantly down-regulated (Fig. 3d). We presumed that this down-regulation might be regulated by HCG11, which was aberrantly highly expressed in GC cells. We browsed from starBase database and found putative miR-1276 binding site in the sequence of HCG11. To determine whether HCG11 bind to the putative binding site, we mutated this sequence accordingly, as illustrated in Additional file 1: Figure S1C. Subsequently, dual luciferase reporter assays were conducted in AGS and BGC-823 cells to study the physical interaction. It was indicated that miR-1276 mimics could markedly attenuate the luciferase activity of HCG11-WT, while no significant alternation in that of HCG11-MUT (Additional file 1: Figure S1D). RIP assay was conducted and the results manifested that HCG11, miR-1276 and CTNNB1 were both enriched in the Ago2 group, suggesting that they all existed in the RNA induced silencing complex (Fig. 3e). In RNA pull down assay, miR-1276 was biotinylated to absorb HCG11 and CTNNB1. The results depicted that HCG11 bind to miR-1276 while CTNNB1 bind to miR-1276 (Fig. 3f). Luciferase reporter assays were performed to illustrate the competing relationship among HCG11, miR-1276 and CTNNB1. The results delineated that the activity of pmirGLO vector built with CTNNB1-WT was dramatically decreased by miR-1276 mimics. After transfecting HCG11 into the cells, the activity was recovered (Fig. 3g). The expression of miR-1276 was distinctly decreased by miR-1276 inhibitor. The Wnt signaling activity was decreased by silencing HCG11, but increased significantly again after co-transfection with miR-1276 inhibitor in AGS and BGC-823 cells through dual luciferase reporter assays (Fig. 3h). The results of western blot assay revealed that the protein of CTNNB1 reduced by sh-HCG11#1 was offset by miR-1276 inhibitor (Fig. 3i) In a word, HCG11 could sponge miR-1276 to regulate CTNNB1.

### HCG11 promoted the proliferation and migration via targeting miR-1276/CTNNB1 in GC

**Fig. 4:**
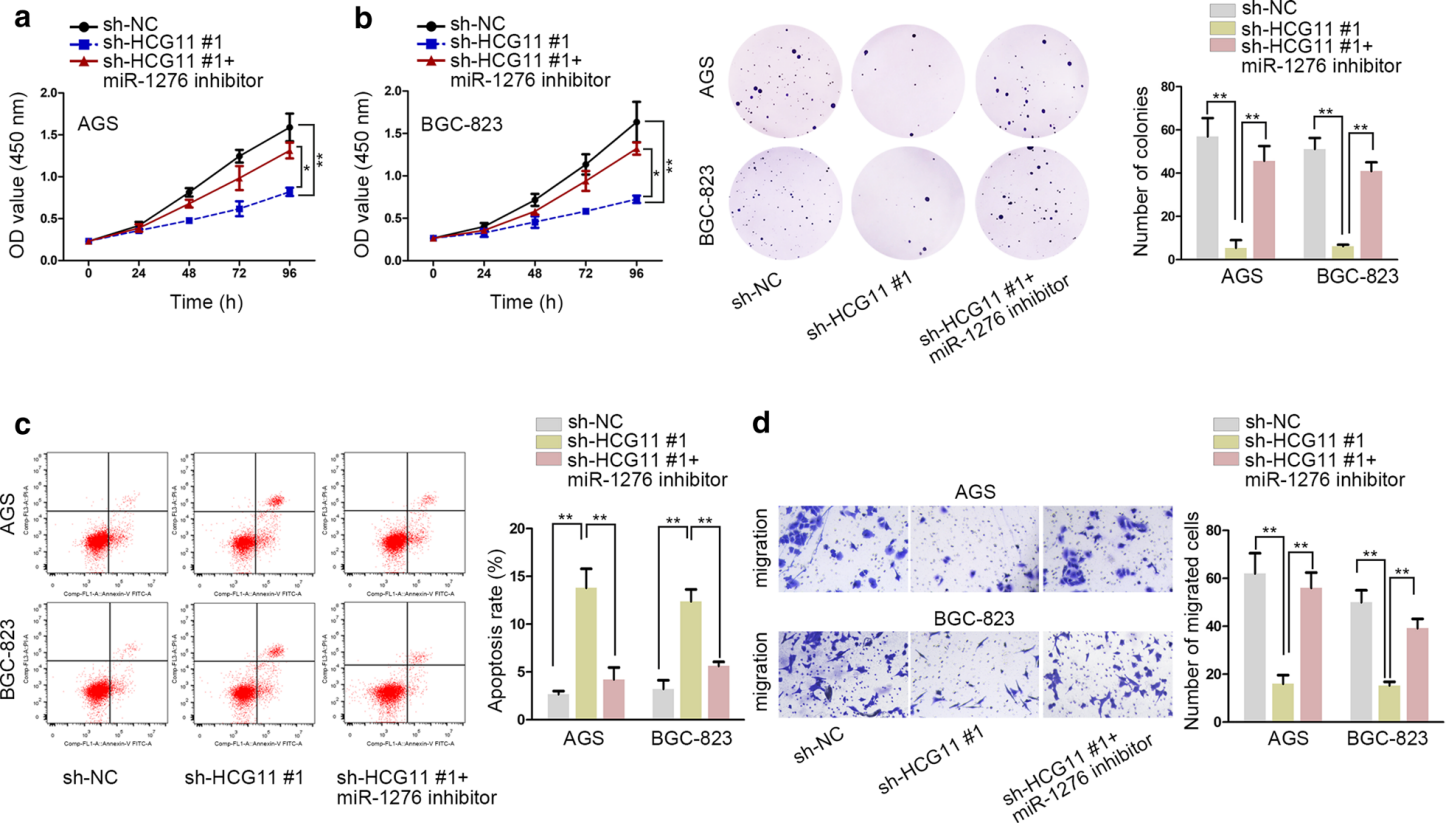
HCG11 promoted the proliferation and migration via targeting miR-1276/CTNNB1 in GC. **a**, **b** CCK8 and colony formation assay were used to evaluate GC cell proliferation ability and knockdown of HCG11 inhibited the ability of cell proliferation, which was regained by miR-1276 inhibitor. **c** The rate of apoptosis was examined by flow cytometry analysis and silencing HCG11 could repress the rate, which was counteracted by miR-1276 inhibitor. **d** The capacity of migration was examined by transwell assay. The suppressing cell migration ability exerted by sh-HCG11#1 was regained by miR-1276 inhibitor. *P < 0.05, **P < 0.01

To verify the effectiveness of HCG11/miR-1276/CTNNB1 axis in GC, the following rescue assays were carried out. The ability of cell proliferation was weakened by sh-HCG11#1 compared with normal control group evaluated by CCK8 and colony formation assay. Interestingly, after both cells transfecting with miR-1276 inhibitor, the cell proliferation capacity was partially regained (Fig. 4a, b) The capacity of apoptosis detected by flow cytometry analysis was a lot higher transfected with sh-HCG11#1 in comparison with the normal control group. The effect caused by sh-HCG11 on apoptosis was counteracted by miR-1276 inhibitor (Fig. 4c). The outcome of transwell assay demonstrated that sh-HCG11#1 could inhibit GC cell migration capacity. However, after transfecting miR-1276 inhibitor into both cells, the mediated-results made by HCG11 silence were restored (Fig. 4d). In conclusion, HCG11 could accelerate cell proliferation and migration via targeting miR-1276/CTNNB1.

### Silencing HCG11 repressed the cell proliferation in vivo

**Fig. 5:**
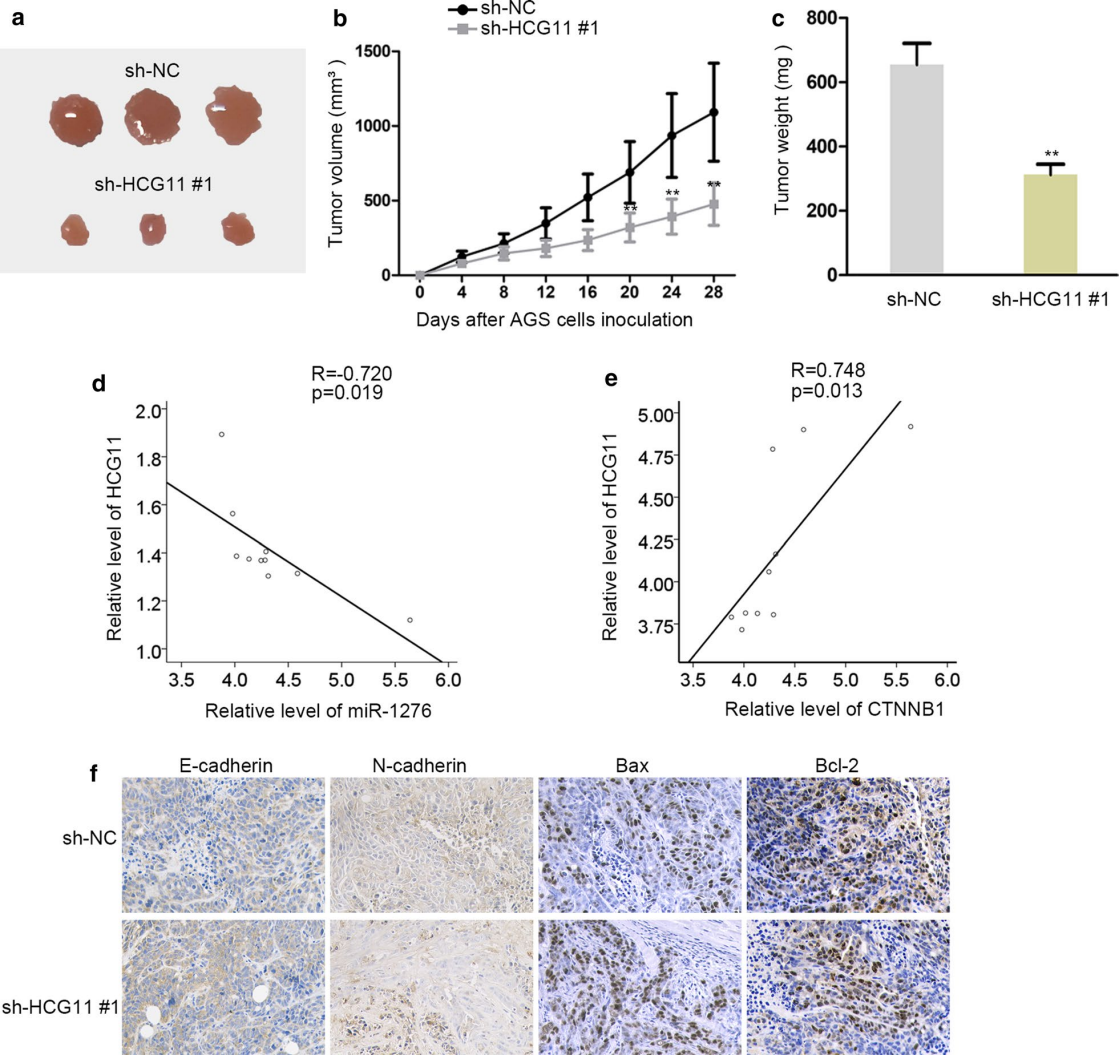
Silencing HCG11 repressed the cell proliferation in vivo. **a** Tumor size was measured and sh-HCG11#1 reduced the tumor size compared with normal control. **b** Tumor volume was measured and sh-HCG11#1 reduced the tumor volume compared with normal control. **c** Tumor weight was measured and sh-HCG11#1 reduced the tumor weight compared with normal control. **d** The correlation of HCG11 and miR-1276 was analyzed by Spearman’s correlation analysis. **e** The correlation of HCG11 and CTNNB1 was analyzed by Spearman’s correlation analysis. **f** Immunohistochemistry assay was performed to detect the level of proteins related to epithelial-mesenchymal transition and apoptosis process. **P < 0.01

To further learn the role of HCG11, we made experiments on nude mice. AGS cells transfected with sh-HCG11#1 were injected into nude mice. As shown in Fig. 5a, silencing HCG11 could suppress the tumor size. The volume of tumor transfected with sh-HCG11#1 was much smaller compared with control group (Fig. 5b). The similar result could be found in tumor weight (Fig. 5c). These samples were used to analyze the correlation among HCG11, mir-1276 and CTNNB1. We found that HCG11 was negatively correlated with miR-1276 and had positive correlation with CTNNB1 (Fig. 5d, e). Overall, HCG11 promoted tumor growth in vivo.

## Discussion

GC is considered as a high-death rate cancer which is hard to cure. Traditional therapies suffered the patients with little effective results. Recently, emerging evidence suggested that lncRNAs had a tight relationship with the occurrence and development of cancers [18, 19]. Aberrantly expressed lncRNAs could function as anti-tumor or tumor promoter in the process of cancers [20–22]. HCG11 was studied in prostate cancer [23], glioma [24], and hepatocellular carcinoma [25]. Although HCG11 functioned as a tumor inhibitor in these studies, the results of our present study showed that HCG11 was high expressed in GC cells and tissues. This result may be caused by tumor tissue specificity. Moreover, silencing HCG11 could hinder the proliferation, migration yet promoted apoptosis in GC cells. Subsequently, we used western blot to evaluate the protein of several common pathways transfected with sh-HCG11#1. The results manifested that HCG11 was involved in activating Wnt signaling pathway in GC cells. As we know that the target mRNA of β-catenin is CTNNB1, so HCG11 acted as a ceRNA to regulate the expression of CTNNB1.

Mounting researches indicated that lncRNA acted as a sponge for miRNA [26–28]. In the current study, 19 miRNA was selected out on condition that it can both bind to HCG11 and CTNNB1. RT-qPCR assay was applied to test the expression of these miRNAs in GC cell lines and tissues. Finally, miR-1276 was selected out. Then, the results of RNA pull down assay revealed that HCG11 could bind to miR-1276 and CTNNB1 could bind to miR-1276. Luciferase reporter assays were conducted to demonstrated competing relationship among HCG11, miR-1276 and CTNNB1. Furthermore, miR-1276 inhibitor could recover the effect caused by knockdown of HCG11 on cell proliferation, migration and activity of Wnt signaling pathway.

CTNNB1 was a kind of mRNA which could translate into protein connected with Wnt signaling pathway [29]. In our investigation, we found that HCG11 acted as a ceRNA to regulate CTNNB1. Protein expression of CTNNB1 was reduced by sh-HCG11#1, after transfecting with miR-1276 inhibitor, CTNNB1 protein was partially recovered. Moreover, the data collected from nude mice experiments delineated that CTNNB1 was positively correlated with HCG11.

## Conclusion

Collectively, the results of our current study showed that HCG11 was highly expressed in GC and knockdown of HCG11 inhibited cell proliferation, migration and the activation of Wnt signaling pathway in GC. HCG11 promoted GC progression by targeting miR-1276/CTNNB1.

## Supplementary information

**Additional file 1: Figure S1.** (A) RT-qPCR analysis was conducted to detect the expression of HCG11 in patients at different stages of gastric cancer. (B) Western blot assay was performed to measure the expression level of proteins related with epithelial-mesenchymal transition after silencing HCG11. (C) The putative miR-1276 binding site in the sequence of HCG11 was predicted by starBase, and the mutate sequence was shown accordingly. (D) Dual luciferase reporter assays were conducted in GC cells to study the mutual interaction between miR-1276 and HCG11. **P < 0.01.

## Data Availability

Research data are not shared.

## Abbreviations

GC: gastric cancer
LncRNA: long non-coding RNA
ceRNA: competing endogenous RNA
miRNA: Micro RNA
