# Supplementary figures and images for: LncRNA HCG11 promotes proliferation and migration in gastric cancer via targeting miR-1276/CTNNB1 and activating Wnt signaling pathway
Source: Cancer Cell Int. 2019 Dec 26;19:350. doi: 10.1186/s12935-019-1046-0 (PMC6933929; doi:10.1186/s12935-019-1046-0)

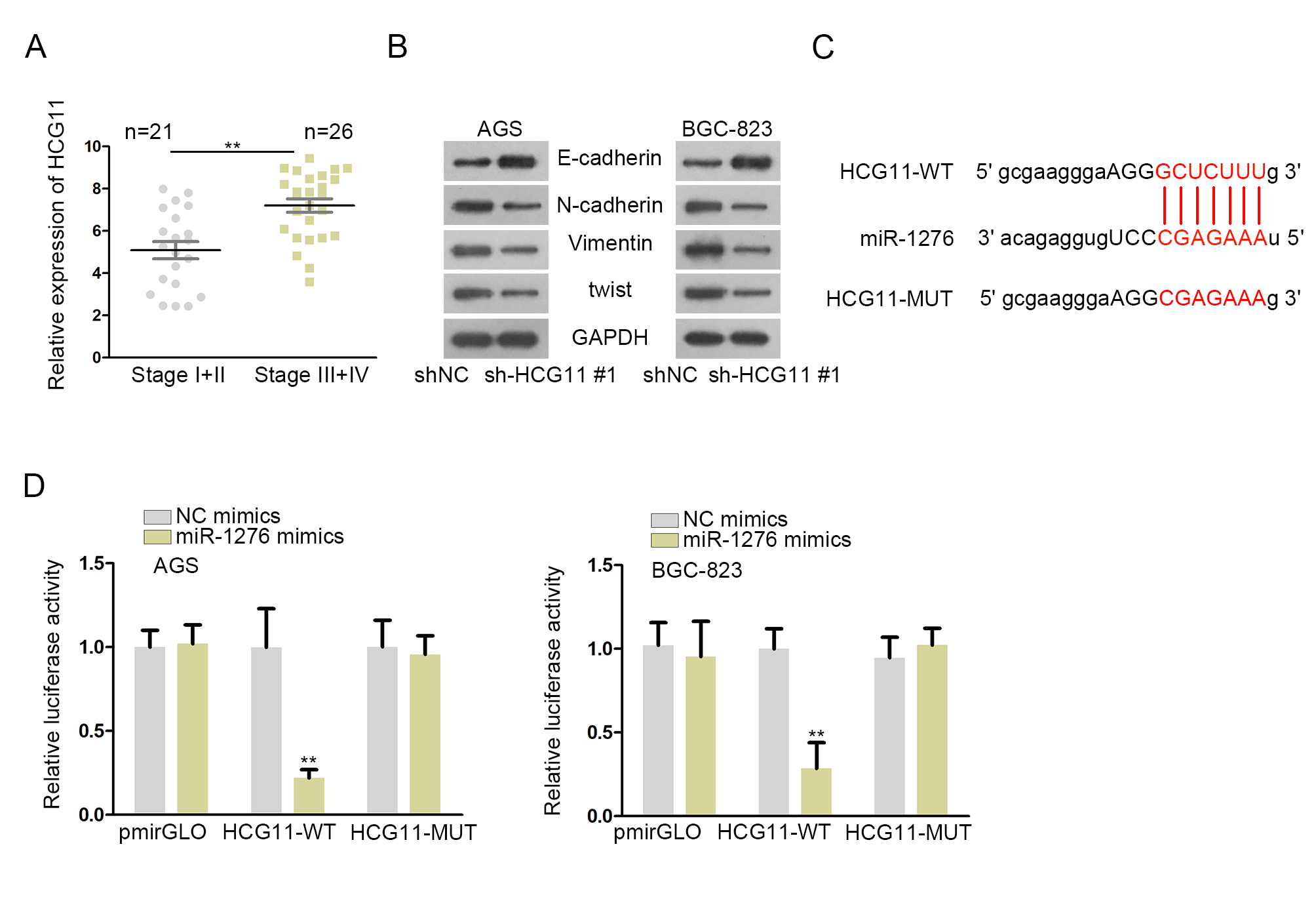

Supplement: Supplementary file 1 — Additional file 1: Figure S1. (A) RT-qPCR analysis was conducted to detect the expression of HCG11 in patients at different stages of gastric cancer. (B) Western blot assay was performed to measure the expression level of proteins related with epithelial-mesenchymal transition after silencing HCG11. (C) The putative miR-1276 binding site in the sequence of HCG11 was predicted by starBase, and the mutate sequence was shown accordingly. (D) Dual luciferase reporter assays were conducted in GC cells to study the mutual interaction between miR-1276 and HCG11. **P < 0.01. [file 12935_2019_1046_MOESM1_ESM.tif]
